# Distribution, Sources, and Health Risk of Short-, Medium- and Long-Chain Chlorinated Paraffins in School-Area Ambient PM_1_: A Study from the Pearl River Delta, China

**DOI:** 10.3390/toxics13060467

**Published:** 2025-05-31

**Authors:** Mo Yang, Xin-Feng Wang, Jing-Wen Huang, Nan-Xiang Jin, Chu Chu, Guo-Feng Huang, Duo-Hong Chen, Min Xie, Yu-Hong Zhai, Yu-Jun Lin, Jun Liu, Li-Zi Lin, Wen-Wen Bao, Zhao-Huan Gui, Pasi I. Jalava, Guang-Hui Dong, Marjut Roponen

**Affiliations:** 1 Inhalation Toxicology Laboratory, Department of Environmental and Biological Science, University of Eastern Finland, Yliopistonranta 1, P.O. Box 1627, FI-70211 Kuopio, Finland; moyang@uef.fi (M.Y.); chuch3@mail2.sysu.edu.cn (C.C.); pasi.jalava@uef.fi (P.I.J.); marjut.roponen@uef.fi (M.R.); 2 Guangdong Provincial Engineering Technology Research Center of Environmental Pollution and Health Risk Assessment, Department of Occupational and Environmental Health, School of Public Health, Sun Yat-sen University, Guangzhou 510080, China; huangjw98@mail2.sysu.edu.cn (J.-W.H.); linlz@mail.sysu.edu.cn (L.-Z.L.); baoww5@mail.sysu.edu.cn (W.-W.B.); guizhh3@mail.sysu.edu.cn (Z.-H.G.); 3 Environment Research Institute, Shandong University, Qingdao 266237, China; xinfengwang@sdu.edu.cn; 4 A.I.Virtanen Institute for Molecular Sciences, University of Eastern Finland, Neulaniementie 2, FI-70210 Kuopio, Finland; nanxiang.jin@uef.fi; 5 Guangdong Ecological Environmental Monitoring Center, Environmental Key Laboratory of Regional Air Quality Monitoring, Ministry of Ecology and Environment, Guangdong Environmental Protection Key Laboratory of Atmospheric Secondary Pollution, Guangzhou 510308, Chinaduohongchen@139.com (D.-H.C.); xieminsysu@163.com (M.X.); zhaiyuhong411@163.com (Y.-H.Z.); linyujun02@126.com (Y.-J.L.); 13798090760@139.com (J.L.); 6 Joint International Research Laboratory of Environment and Health, Ministry of Education, Guangdong Provincial Engineering Technology Research Center of Environmental Pollution and Health Risk Assessment, Department of Preventive Medicine, School of Public Health, Sun Yat-sen University, 74 Zhongshan 2nd Road, Yuexiu District, Guangzhou 510080, China

**Keywords:** chlorinated paraffins, ambient PM_1_, source, children, health risk

## Abstract

Background: Only a few studies have reported on chlorinated paraffin (CP) levels, especially long-chain chlorinated paraffins (LCCPs), in submicron particulate matter (PM_1_) in the outdoor air around primary and secondary schools. Methods: This study examined concentrations of short-chain CPs (SCCPs), medium-chain CPs (MCCPs), and LCCPs in PM_1_ samples from 96 schools across six cities in China’s Pearl River Delta region during the winter (October to December 2018). Results: The median total CP concentration was 34 ng/m^3^, with median values for SCCP, MCCP, and LCCP of 17.3, 15, and 0.7 ng/m^3^, respectively. The primary congeners were C_13_Cl_6–8_ for SCCPs, C_14_Cl_6–9_ for MCCPs, and C_18_Cl_7–10_ for LCCPs. The SCCPs and MCCPs largely originated from fugitive dust, whereas the LCCPs were mainly sourced from organic chemical industries. Air masses from the South China Sea contributed most to SCCP and MCCP levels, while those from the east coast accounted for the highest LCCP levels. The concentrations of CP in PM_1_ were significantly positively correlated with PM_1_ levels. Conclusions: The exposure risk assessments by age indicated a very low current health risk from PM_1_-related CP inhalation, although prolonged pollution could raise these risks as CP concentrations in ambient PM may continue to increase.

## 1. Introduction

Chlorinated paraffins (CPs), a class of synthetic chemicals primarily used as flame retardants, plasticizers, and lubricants [1,2], have attracted increasing concern due to their persistence, bioaccumulation potential, and toxicity to both humans and the environment [3,4]. CPs can be categorized into short-chain (C_10_–C_13_, SCCPs), medium-chain (C_14_–C_17_, MCCPs), and long-chain chlorinated paraffins (C_18_–C_30_, LCCPs) based on their carbon chain lengths [5]. These compounds are released into the atmosphere during various stages of their production, use, and disposal [6,7]. Once in the atmosphere, they eventually bind to particulate matter (PM) due to their low vapor pressure and high hydrophobicity [8]. Therefore, they can be transported over long distances through the atmosphere, deposited in various environmental compartments, and potentially inhaled by humans [8,9].

China is a major global producer and consumer of CPs [7,10], with the Pearl River Delta (PRD) region being one of the country’s most industrialized and polluted regions [11]. The PRD region experiences high levels of pollutants such as PM_2.5_ (with an aerodynamic diameter ≤ 2.5 µm) and PM_1_ (with an aerodynamic diameter ≤ 1 µm), which can act as carriers for toxic chemicals including CPs. PM_1_ is especially concerning for public health as it can penetrate deeply into the respiratory system and pose serious health risks [12,13]. It has been suggested that PM_1_ could serve as a more effective measure of air quality and potential health risks than PM_2.5_ [14]. Although numerous studies have investigated the distribution of CPs in larger particulate fractions such as PM_2.5_ in the PRD [15,16] and other regions [17,18,19], there is limited information on CP concentrations in PM_1_ [20,21].

Understanding CP levels in ambient air pollution is crucial in environments where vulnerable populations, such as children, are exposed. Children are more susceptible to air pollution due to their developing respiratory systems and higher rates of air intake per body weight compared to adults [22]. Schools, where children spend much of their time, are crucial locations for evaluating air quality, especially in regions with high industrial pollution such as the PRD. However, research on CP exposure in schools, particularly regarding fine PM and its smaller subfraction PM_1_, remains limited.

Our previous research has identified significant concentrations of CPs in PM_2.5_ in the PRD region, highlighting their atmospheric transport potential and human exposure risks [15]. This study extends those findings to investigate the presence of CPs in PM_1_, focusing on primary and secondary schools from six cities in the PRD region during the winter of 2018. The winter season is particularly relevant as lower temperatures and meteorological conditions can trap pollutants near the surface, leading to increased pollutant levels [23]. We analyzed the sources of CPs in PM_1_ and also performed a back-trajectory analysis to examine the origin and transport pathways of the air masses reaching the study areas during the sampling period. This combined approach allows for a comprehensive understanding of both local and regional contributions to CP pollution in school-area environments. This paper aims to address the gap in knowledge regarding CP concentrations in PM_1_ in school environments, providing critical insights into the potential exposure risks for children in the PRD region.

## 2. Materials and Methods

### 2.1. Sample Collection

PM_1_ samples were collected from outdoor air at 96 primary and secondary schools within 1 km of municipal monitoring stations across six cities in the PRD region (Guangzhou, Foshan, Shenzhen, Zhuhai, Zhongshan, and Maoming), covering both inland and coastal areas as well as urban and suburban settings, between October and December 2018. In each administrative district of these cities, we ensured the inclusion of at least one primary and one secondary school, allowing us to capture spatial heterogeneity across different microenvironments and population groups. A medium-volume sampler (TH150C/D, Tian Hong Instrument Co., Ltd., Wuhan, China) operating at 100 L/min for 24 h was used, collecting approximately 150 m^3^ of air per site. Quartz fiber filters (Whatman Inc., Cytiva, Maidstone, UK) were used, and samplers were calibrated before each use. Field blank samples were exposed to ambient air for 0.5 min to assess and remove sampling background contamination. After sampling, filters were wrapped in aluminum foil and stored at −20 °C until analysis.

### 2.2. Sample Analysis

All chemicals and reagents used are listed in Table S1. Sample pretreatment followed Huang et al. 2023 [15]. Briefly, one-fourth of each quartz filter was spiked with 5 ng of ^13^C_10_-trans-chlordane and extracted twice using dichloromethane/n-hexane (1:1, *v*/*v*) in an ultrasonic bath (<20 °C, 20 min). The combined extract was concentrated to 2 mL under nitrogen and purified using a multilayer column (Florisil, silica gels, and Na_2_SO_4_). After evaporation to near dryness, samples were re-dissolved in 100 μL methanol with ^13^C_6_-triclocarban and analyzed by UPLC-QTOF-MS (X500R, Sciex, Concord, ON, Canada) using an ACQUITY BEH Shield RP18 column. A water/methanol gradient with 10 mM ammonium acetate was used at 0.4 mL/min. MS was run in negative full scan mode (*m*/*z* 180–1200) with mass accuracy within 5 ppm. Since ^13^C_10_-trans-chlordane could not be quantified by UPLC-QTOF-MS, its recovery was determined by GC-MS/MS (Agilent 7890B-7000D, Agilent Technologies Inc., Santa Clara, CA, USA). After UPLC analysis, solvents were replaced with hexane and spiked with ε-hexachlorocyclohexane before GC-MS/MS.

### 2.3. Identification and Quantification of CPs

The UPLC-QTOF-MS monitored [M–H]⁻ ions, selecting the two most abundant isotope clusters for qualitative and quantitative analysis. In total, 36 SCCP (C_10_C_l4_–C_13_Cl_12_), 40 MCCP (C_14_Cl_4_–C_17_Cl_13_), and 115 LCCP (C_18_Cl_4_–C_27_Cl_15_) congener groups were analyzed (Tables S2 and S3). Congeners were identified based on [M–H]⁻ mass accuracy (<10 ppm), retention times, and isotope ratios compared to standards. Quantification followed Reth et al. 2025 [24], using linear correlations between chlorination degree and total response factors (R^2^ > 0.9 for all CP types).

### 2.4. Quality Control and Assurance

Laboratory (n = 23) and field blanks (n = 14) were analyzed alongside PM_1_ samples, with one procedural and one lab blank per 10-sample batch. Mean (±SD) blank concentrations were 4.3 ± 1.8 ng (SCCPs), 4.6 ± 2.5 ng (MCCPs), and 1.1 ± 0.87 ng (LCCPs). Sample concentrations were blank-corrected. Recoveries of ^13^C_10_-trans-chlordane ranged from 52.4% to 91.6%. In six spiked blank filters (50 ng standard), recoveries were 83–110% (SCCPs), 85–107% (MCCPs), and 70–135% (LCCPs). Limits of quantification (LOQs), set at 10× the blank SD, were 1.26 ng/m^3^ (SCCPs), 1.02 ng/m^3^ (MCCPs), and 0.35 ng/m^3^ (LCCPs).

### 2.5. Source Apportionment with PMF Model

A positive matrix factorization (PMF) model was used to identify major sources of chlorinated paraffins in submicron particles and estimate their contributions. The model included 20 variables: SCCPs, MCCPs, LCCPs, 8 water-soluble ions (e.g., sulfate, nitrate), organic and elemental carbon, and 7 trace elements (e.g., Fe, Zn, Pb). Data from 89 samples with complete measurements were used for the analysis. The required data of concentrations and uncertainty were determined as follows [25].

For concentration values less than the detection limits:

(1) $$x_{ij}=\frac{{DL}_{i}}{2},\sigma_{ij}=\frac{5{DL}_{i}}{6}$$

For concentrations more than the detection limits:

(2) $$x_{ij}=c_{ij}$$

(3) $$\mathrm{If}\;x_{ij}\leq 3{DL}_{i},\;\sigma_{ij}=\frac{{DL}_{i}}{3}+0.2\times c_{ij};$$

(4) $$\mathrm{If}\;x_{ij}>3{DL}_{i},\;\sigma_{ij}=\frac{{DL}_{i}}{3}+0.1\times c_{ij};$$

where $x_{ij}$ is the concentration value of the species i for the sample j; ${DL}_{i}$ is the detection limit of the species I; $\sigma_{ij}$ is the uncertainty value corresponding to the concentration $x_{ij}$; and $c_{ij}$ is the measured concentration.

Source factors were identified based on chemical profiles and time series comparisons with tracers. Models with 4–8 factors were tested, and seven factors were selected as optimal based on Q/Qexp, residual analysis, bootstrap variability, and source interpretability. The predicted concentrations closely matched observed data (Figure S1).

### 2.6. Backward Trajectory Analysis

The site coordinates, average air pressure, and ambient temperature for the sampling sites are detailed in Table S4. To analyze the effects of varying air masses on CP levels and compositions, 24 h backward trajectories were calculated for all samples using the HYSPLIT model, available through the NOAA Air Resources Laboratory (https://www.ready.noaa.gov, accessed on 10 October 2024) [26,27]. Trajectories were generated with meteorological data from GDAS, set at an altitude of 100 m above ground, with a six-hour interval between calculations. It should be noted that the HYSPLIT back-trajectory analysis is subject to certain uncertainties, especially in coastal regions such as the Pearl River Delta. Variability in boundary layer dynamics, sea–land breeze circulations, and the resolution of meteorological data can affect the accuracy of calculated air mass trajectories. Therefore, the results should be interpreted as indicative of general transport patterns rather than precise source allocations.

### 2.7. Exposure Risk Assessment

The assessment of exposure risk to CPs through inhalation of PM_1_ involved the calculation of estimated daily intake (EDI, ng/kg/day), hazard quotient (HQ, unitless), and margin of exposure (MOE, unitless). Each sampling site was individually assessed for EDI, HQ, and MOE using the following equations: Equation (1): EDI = (C × IR × T)/BW, Equation (2): HQ = EDI/TDI, and Equation (3): MOE = NOAEL/EDI. The concentration of CPs in PM_1_ (C, ng/m^3^) was determined. The inhalation rate (IR, m^3^/day) and body weight (BW, kg) were obtained from the Chinese Exposure Factors Handbook (adult) [28] and the Chinese Exposure Factors Handbook (children) [29], as summarized in Table S5. The average exposure time per day (T, hour/day) was assumed to be 12 h (0.5 d). The tolerable daily intakes (TDIs) for SCCPs, MCCPs, and LCCPs for non-neoplastic effects were all 100 μg/kg/day [30]. The non-observed adverse-effect levels (NOAELs) of SCCPs, MCCPs, and LCCPs were reported as 10, 23, and 100 mg/kg/day, respectively [28,30].

### 2.8. Statistical Analysis

Concentrations of samples below the LOQs were assigned as the value of the LOQ/√2 in the statistical analysis. The Shapiro–Wilk test was used to test the normality of the data. For the non-normal distributions of CP concentrations in samples, the Kruskal–Wallis test and subsequent post hoc pairwise test (with Bonferroni adjustment) were used to explore the differences among cities. Spearman’s rank correlation was applied to evaluate the relationships between CP concentrations and their correlations with other components in PM_1_.

## 3. Results and Discussion

### 3.1. Concentrations and Spatial Variations of CPs in PM_1_

The concentrations of CPs in the ambient PM_1_ from the primary and secondary schools of the six cities in the PRD region are summarized in Table 1. The SCCPs, MCCPs, and LCCPs showed detection rates exceeding 90%. This suggests that CPs are broadly present in environmental PM throughout the PRD region. The concentration of total CPs (ΣCPs) in PM_1_ varied between 14.1 and 181.9 ng/m^3^, with a median value of 34 ng/m^3^. Specifically, the median concentrations for ΣSCCPs, ΣMCCPs, and ΣLCCPs were 17.3, 15, and 0.7 ng/m^3^, respectively. SCCPs contributed an average of 53.9% to the total CPs, MCCPs accounted for 43.5%, and LCCPs made up 2.6%, indicating SCCPs and MCCPs as the dominant contributors. All the levels of SCCPs, MCCPs, and LCCPs in PM_1_ from each sampling site are presented in Figure S2. The contamination from CPs was significantly higher than that of other common atmospheric pollutants, such as polychlorinated dibenzo-dioxins/furans and polybrominated diphenyl ethers, with concentrations reaching the ng/m^3^ range [31,32]. The concentrations of SCCPs and MCCPs in PM_1_ from this study were close to those in most particle phases and PM_2.5_ from Beijing [33], communities in 9 cities in the PRD [16], and 10 cities in China [34], but were lower than those in PM_2.5_ from Shandong, Jinan [35], and higher than those in Henan [36] and Dalian [19]. The detailed data comparison is shown in Table S6. The concentrations of SCCPs and MCCPs observed in PM_1_ were higher than in PM_2.5_ (SCCPs median: 6.5 ng/m^3^, range: 0.8–32.1 ng/m^3^; MCCPs median: 6.3 ng/m^3^, range: 1.0–24.1 ng/m^3^) [15] collected in the winter during the same period and at the same site, but the concentration of LCCPs in PM_1_ was lower than that in PM_2.5_ (median: 1.4 ng/m^3^, range: 0.2–13.0 ng/m^3^). One of the main reasons may be the flow rate of the sampler. In this study, the PM_1_ sampler had a flow rate of 0.1 m^3^/min, while the PM_2.5_ sampler operated at 1.05 m^3^/min [15]. Due to the higher flow rate, the high-flow sampler lost some CPs during the sampling process.

In China, two main methods are used to produce commercial CPs: the bulk (solvent) process and the aqueous process [37]. The bulk method, suitable for a wide range of alkane chain lengths, directly chlorinates feedstock and is widely used in the PRD region, where demand for SCCPs is high [38]. Common SCCP products such as CP-42 and CP-52, known for their moderate chlorine content and fluid properties, are preferred for applications requiring flame retardancy and durability, likely contributing to the significantly higher SCCP levels observed in this study compared to MCCPs and LCCPs (*p* < 0.05). In contrast, the aqueous method—more suitable for longer chains—is mainly used in eastern provinces such as Jiangsu and Zhejiang, where MCCPs and LCCPs are favored for use in plasticizers and other specialized materials. Notably, the growing use of MCCPs in the PRD is evident from the increasing MCCP/SCCP ratio found in marine mammals from the South China Sea [39]. Moreover, some studies have reported higher MCCP levels than SCCPs in outdoor PM_2.5_ in the region during the summer months [16]. Such variations could be attributed to environmental transport, differences in sampling timeframes, geographic coverage, and analytical techniques.

In this study, the elevated levels of CPs observed during the sampling period (winter) may be influenced by winter-specific meteorological conditions in the PRD region. Cooler temperatures, lower atmospheric boundary layers, and more stable weather conditions during the winter months tend to reduce atmospheric dispersion, leading to the accumulation of PM and associated pollutants such as CPs [35]. In addition, increased biomass burning and heating-related emissions during the colder months can also contribute to higher CP concentrations [31]. It is likely that the concentration levels and atmospheric behavior of CPs would differ in warmer seasons (e.g., spring or summer), when enhanced photochemical activity and stronger vertical mixing may promote the degradation and dispersion of CPs. Further seasonal sampling would be necessary to confirm these trends and to understand the full seasonal dynamics of CPs in PM_1_.

Zhuhai recorded the highest median concentration of total CPs in PM_1_ (45.2 ng/m^3^; range: 18.5–59.7 ng/m^3^), followed by Maoming, Guangzhou, Shenzhen, Zhongshan, and Foshan, as shown in Table 1. However, the statistical analysis revealed no significant variation in CP levels among the six cities (Figure S3). The relatively elevated levels in Zhuhai may be linked to local industrial operations, particularly in the chemical sector, while differences in emission sources and weather patterns likely contributed to the spatial variation observed across the region.

The widespread presence of CPs in PM_1_ in the PRD region underscores the pressing need for targeted pollution control strategies. As a major economic hub, the PRD’s industrial and transportation activities contribute significantly to CP emissions, which may have far-reaching impacts on regional air quality. Notably, the high levels of SCCPs and MCCPs observed in this study indicate that these pollutants could be transported over long distances, potentially affecting neighboring regions and marine ecosystems in the South China Sea. This highlights the importance of implementing stricter regulations on CP emissions, especially in industrial cities such as Zhuhai and Maoming, and adopting cleaner production technologies to mitigate environmental impacts.

### 3.2. Carbon and Chlorine Congener Group Profiles of CPs in PM_1_

**Figure 1:**
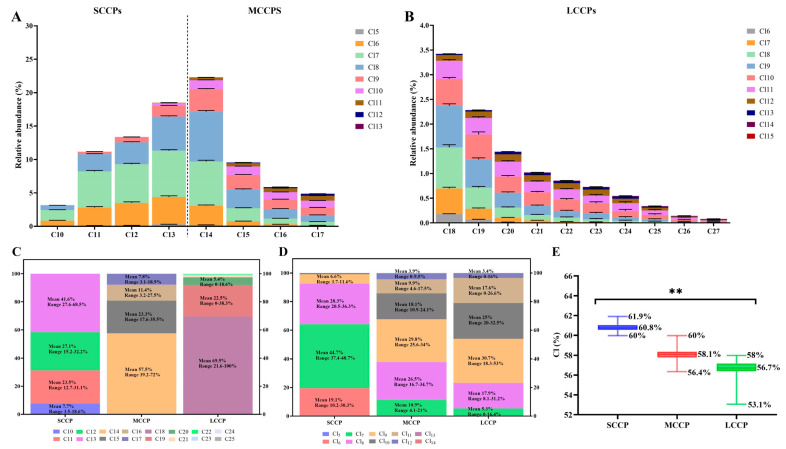
(**A**,**B**) Relative abundance profiles of congener groups for SCCPs, MCCPs, and LCCPs in PM_1_ samples collected from the Pearl River Delta region, China. The profiles are grouped based on carbon chain length and number of chlorine atoms. (**C**) Distribution of alkane chains (C_10_–C_13_ for SCCPs, C_14_–C_17_ for MCCPs, and C_18_–C_30_ for LCCPs). Data are presented as mean and range, indicating the relative contribution (%) of each chain length within its respective CP class. (**D**) Chlorine content (%) of SCCPs, MCCPs, and LCCPs, shown as mean and range. (**E**) Chlorination level (average number of Cl atoms per molecule) for each CP class, highlighting the degree of chlorination. Asterisks (**) indicate statistically significant differences between groups (*p* < 0.001).

The relative abundance of congener group profiles of SCCPs, MCCPs, and LCCPs in PM_1_ are shown in Figure 1A,B. The predominant alkane chain length of the SCCPs was C_13_, comprising 27.6% to 68.5% (mean 41.6%), with C_12_ (mean 27.1%), C_11_ (mean 23.5%), and C_10_ (mean 7.7%) following (Figure 1C). For the MCCPs, C_14_ was the most abundant, ranging from 39.2% to 72% (mean 57.5%), followed by C_15_ (mean 23.3%), C_16_ (mean 11.4%), and C_17_ (mean 7.8%) (Figure 1C). The CP homolog patterns in the ambient atmosphere were dominated by C_10–14_ compounds, showing a strong similarity to those observed in the CP products, as well as in indoor air and dust samples [40,41]. In commercial mixtures such as CP-42, CP-52, and CP-70 [33], the C_13_ congener group is dominant [42], while in domestic polymer products, C_11_ and C_13_ homologs are the primary carbon groups [43]. For the LCCPs, C_18_ dominated (mean 69.5%), with C_19_ (mean 22.5%) and C_20_ (mean 5.4%) present in lower mean values (Figure 1C).

In terms of chlorine distribution (Figure 1D), Cl_7_ (mean 44.7%) was the main chlorine group in the SCCPs, followed by Cl_8_ (28.3%) and Cl_6_ (19.1%). For the MCCPs, Cl_8_ (29.8%) was the most common, with Cl_7_ (26.5%) and Cl_9_ (18.1%) also present. In the LCCPs, Cl_9_ was the predominant chlorine group (30.7%), followed by Cl_10_ (25%) and Cl_8_ (17.9%). The mean chlorination level was 60.8% for the SCCPs, 58.1% for the MCCPs, and 56.7% for the LCCPs (Figure 1E). In China’s three main commercial CP products, the chlorination levels vary: MCCPs have the highest chlorination in CP-42, followed by SCCPs and LCCPs; in CP-52, SCCPs rank highest, followed by MCCPs and LCCPs; and in CP-70, LCCPs exhibit the highest degree of chlorination, followed by SCCPs and MCCPs [42]. In the PRD region, CPs primarily originate from the extensive usage of commercial mixtures of CP-42 and CP-52 [42]. This likely explains the chlorine pattern, dominated by Cl_6_–Cl_9_, and the chlorination level in SCCPs, MCCPs, and LCCPs observed in both PM_1_ in this study and in the PM_2.5_ samples in a previous study [15].

The observed differences in chlorination levels among the SCCPs, MCCPs, and LCCPs may also have implications for their environmental persistence and toxicity. Previous studies have indicated that CP congeners with higher chlorination degrees generally exhibit greater hydrophobicity, bioaccumulation potential, and resistance to degradation, which may enhance their environmental persistence and health risks [35]. For instance, highly chlorinated CPs have been found to bind more strongly to particulate matter and accumulate in biota. Although our study did not directly assess these effects, the chlorination patterns observed here suggest the presence of CP mixtures with varying environmental behaviors and toxicological profiles, warranting further investigation in future work.

### 3.3. Source of CPs in PM_1_ in the PRD Region

**Figure 2:**
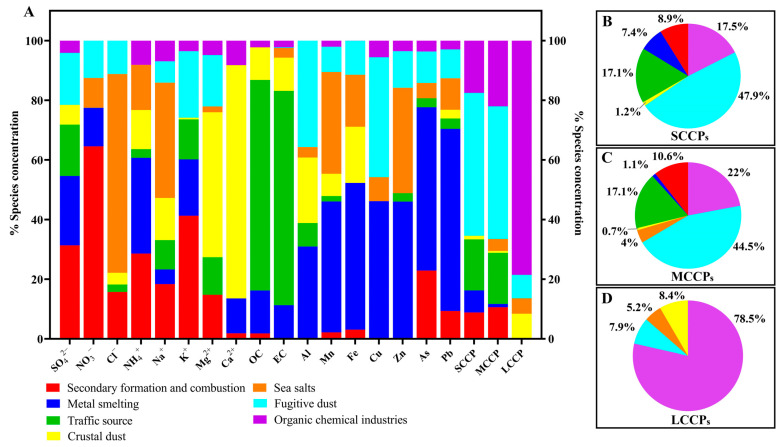
Contributions of secondary formation and combustion, metal smelting, traffic sources, crustal dust, sea salts, fugitive dust, and organic chemical industries to the sources of SCCPs, MCCPs, LCCPs, and other components in PM_1_ from six cities in the Pearl River Delta, China (**A**). (**B**–**D**): The contributions of seven sources to SCCPs, MCCPs, and LCCPs. The data are percentages (%) from different sources.

Seven potential sources of SCCPs, MCCPs, and LCCPs in PM_1_ were identified using specific tracers. These sources included organic chemical industries, fugitive dust, sea salts, crustal dust, traffic sources, and metal smelting, as well as secondary formation and combustion processes, based on the PMF results. As shown in Figure S4, organic chemical industries were characterized by high contributions of LCCPs. Fugitive dust was identified as Al, Cu, K^+^, Mg^2+^, SCCP, and MCCP. Sea salts were marked by high Cl^−^ and Na^+^ levels, while crustal dust was characterized by Mg^2+^ and Ca^2+^. Traffic sources showed high OC and EC concentrations. Metal smelting featured Mn, Fe, Cu, Zn, As, and Pb, and secondary formation and combustion sources were characterized by SO_4_^2−^, NO_3_^−^, NH_4_^+^, K^+^, and As. In this study, the main source of SCCPs and MCCPs was fugitive dust, followed by organic chemical industries and traffic sources (Figure 2A). This high contribution from dust sources aligns with existing research suggesting that CPs may become airborne through the resuspension of soil particles [44]. Organic chemical industries were the main source of LCCPs, accounting for 78.5% (Figure 2A,C). These patterns highlight the differing emission profiles across CP chain lengths, with shorter and mid-chain CPs being more broadly distributed due to resuspension and traffic-related sources, while LCCPs were more closely tied to chemical industries. Such differences underscore the need for targeted control measures across varied sources to address CP pollution in the region.

**Figure 3:**
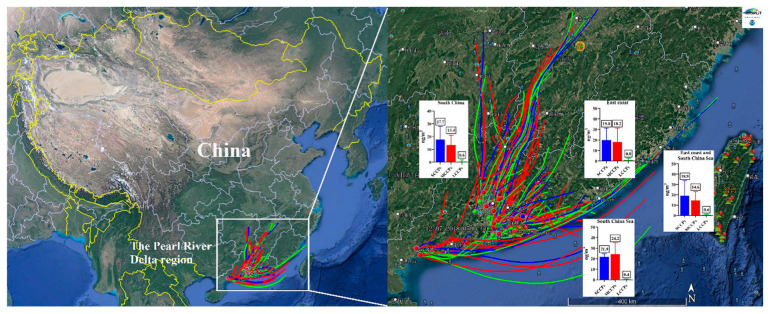
Backward trajectories of air masses and the concentrations of SCCPs, MCCPs, and LCCPs in PM_1_ in the Pearl River Delta, China from different air mass directions. The values in the histograms refer to the concentrations (ng/m^3^) of SCCPs, MCCPs, and LCCPs.

The PRD region, covering southern Guangdong Province, Hong Kong, and Macao, sits along China’s southern coastline and is significantly impacted by the Asian monsoon. This monsoon system influences the climate, air patterns, and environmental conditions of the region. The air mass directions of all the sampling points in the six cities are presented in Figure S5. A backward trajectory analysis indicated that among all the air mass directions (South China, the east coast, and the South China Sea), the South China Sea contributed the highest SCCP and MCCP concentrations (SCCP geometric mean 21.9 ng/m^3^, and MCCP 24.2 ng/m^3^) (Figure 3). The SCCP contributions from these air masses varied slightly (range 17.7–21.9 ng/m^3^), while the MCCP levels showed greater differences (range 13.4–24.2 ng/m^3^), possibly due to specific sources or regional conditions favoring MCCP emissions. Although they had lower LCCP concentrations, air masses from the east coast doubled LCCP levels (0.8 ng/m^3^) compared to those from the South China Sea (0.4 ng/m^3^). This may reflect local variations in CP usage or environmental conditions affecting the CP distribution across regions.

### 3.4. Correlation of CPs and Other Components in PM_1_

The concentrations of SCCPs, MCCPs, and LCCPs in PM_1_ were significantly correlated (*p* ≤ 0.001) (Figure S6). Environmental processes, such as atmospheric deposition and particulate resuspension, can evenly distribute these compounds across particle sizes, maintaining their proportionality in PM_1_. Atmospheric processes such as deposition and resuspension of particulate matter likely contribute to the even distribution of these compounds across different particle sizes, maintaining their proportionality in PM_1_. This shared emission and distribution pattern could drive the observed correlation. The concentrations of the other components in PM_1_ are shown in Figure S7. The concentrations of all types of CPs were significantly correlated with PM_1_ concentrations (*p* ≤ 0.001) (Figure S8). The correlation suggests that these compounds may share a common emission source or follow similar distribution patterns in the environment. This shared emission and distribution pattern could explain the observed correlations. MCCP concentrations were significantly correlated with ion levels, and LCCP concentrations were significantly correlated with ions and metals (Figure S8). The significant correlation of MCCP concentrations with ion levels suggests that MCCPs might be associated with atmospheric chemical processes or secondary aerosol formation, potentially linked to combustion sources. The correlation between LCCP concentrations and both ions and metals further support the idea that LCCPs may be co-emitted with metals from industrial activities or combustion processes. These findings underline the complex interactions between different pollutants in the atmosphere and their potential health implications, particularly as PM_1_ can penetrate deeply into the respiratory system. Future research should focus on source apportionment and toxicity assessments to better understand the environmental and health risks posed by these compounds.

### 3.5. Human Exposure Assessment

**Figure 4:**
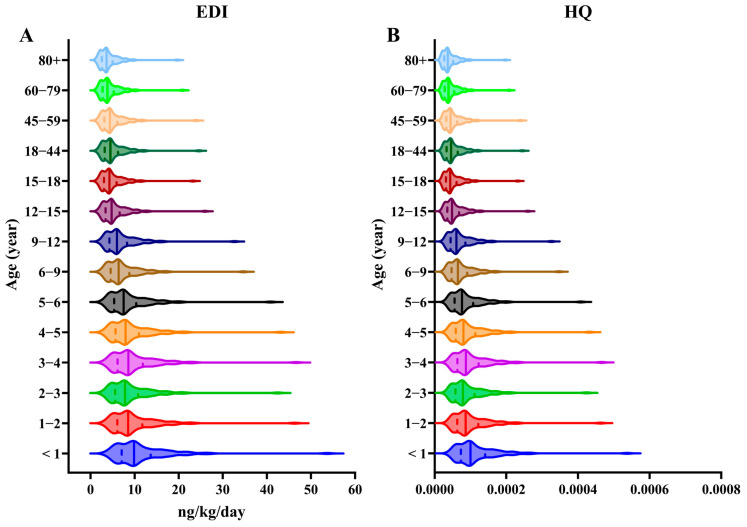
(**A**) Age-specific estimated daily intakes (EDIs) and (**B**) hazard quotients (HQs) of ΣCPs through PM_1_ inhalation. Solid lines indicate the median of each group.

Human exposure to CPs mainly occurs through diet [45], inhalation [9], dust intake [9], and skin contact [46]. Studies indicate that for the general population, dietary intake represents the largest exposure route, accounting for approximately 85% of the total exposure. Inhalation contributes around 15%, while dust intake is considered minimal [47]. Though less significant than diet, inhalation still poses a relevant exposure pathway for the general population, highlighting the importance of air quality in non-occupational CP exposure. We used EDI, HQ, and MOE to evaluate the inhalation exposure risk of CPs to human health. Our study found that infants and young children faced the highest inhalation risk of CPs (with a median EDI of 10.03 ng/kg/day and an HQ of 1 × 10^4^) due to their lower body weight and higher respiratory rate (Figure 4). The EDI ranking across the six cities was Zhuhai > Maoming > Shenzhen > Zhongshan > Guangzhou > Foshan (Figure S9). The EDI and CP concentration rankings for these cities were nearly identical. The HQs across all the age groups were under 1, and the MOEs (Figure S10) for all the CP types exceeded 1000 in our study. These findings suggest that inhalation exposure to PM_1_-associated CPs likely poses minimal or no health risks to humans.

Although our study did not measure CP concentrations in the gas phase or in primary and secondary school indoor environments (e.g., classrooms and homes), which could lead to an underestimation of the inhalation risk, the continuous nature of inhalation exposure remains concerning. Effective ways to reduce inhalation exposure (especially in outdoor air) are currently limited. As CP levels in PM_1_ rise, EDI increases accordingly, which is a potential concern for the PRD and other areas with ongoing pollution.

From a public health perspective, while our study suggests minimal immediate risks from inhalation exposure, the cumulative effects of long-term exposure to CPs in high-pollution areas such as the PRD should not be overlooked. Given the elevated exposure levels among children, schools in heavily polluted regions should consider implementing air quality improvement measures, such as installing air purification systems and increasing green spaces around school premises, to reduce the potential health risks.

The CPs in PM_1_ at primary and secondary schools in the PRD region in the winter primarily consisted of SCCPs and MCCPs, with fugitive dust being their main source. Although the LCCP levels were low, they were widely present in PM_1_ and mainly stemmed from organic chemical industries. Air masses from the South China Sea contributed most to SCCP and MCCP levels, while those from the east coast contributed the highest LCCP levels. The concentrations of all types of CPs in PM_1_ were strongly positively associated with PM_1_ levels. The exposure risk assessments based on HQs and MOEs indicated no significant health risk to the general population from inhaling CPs in PM_1_. However, the EDI increased as the CP concentrations in PM_1_ increased, suggesting that in persistently polluted regions such as the PRD, the potential health risks of CP exposure via inhalation should not be overlooked. Future work will include a comprehensive comparative risk assessment of multiple pollutants in PM_1_ to better contextualize the health risks associated with CPs in school environments.

### 3.6. Strengths and Limitations

This study represents the first large-scale investigation of CPs, including SCCPs, MCCPs, and LCCPs, in submicron PM_1_ in the outdoor air surrounding primary and secondary schools in China’s PRD region. Our study provides comprehensive and regionally representative data that fill a critical gap in the current understanding of CP pollution in school environments. Furthermore, this study explores the sources and atmospheric transport patterns of different CP homologues and evaluates the potential health risks for children by age group, offering a valuable reference for environmental health assessments and air quality management in school settings.

Several limitations should be acknowledged. First, this study focused solely on the concentrations of CPs in the outdoor ambient PM_1_, not the gas-phase CP levels; we also did not simultaneously assess the CP concentrations in the indoor air at schools or at children’s homes. As a result, the overall exposure to CPs via PM_1_ inhalation in children may be underestimated. Second, our sampling was conducted only during the winter season and therefore does not capture seasonal variations in CP levels throughout the year. This limits the generalizability of our findings across different seasons. Third, when comparing CP concentrations in PM_1_ and PM_2.5_, the differences in the sampling flow rates for each particle size fraction may have introduced bias, potentially affecting the accuracy of the comparisons between the two particle size categories.

Our findings have potential implications for environmental health policy and urban planning. The elevated levels of chlorinated paraffins in PM_1_ around the schools suggest a need for targeted monitoring and risk management strategies in educational environments. Since children are more vulnerable to inhalation exposure due to higher respiration rates and developing respiratory systems, policies could prioritize air quality standards specifically for school zones, especially those located near industrial sources. Furthermore, urban planners could consider incorporating buffer zones between industrial operations and residential or school areas to minimize exposure risks. These results also underscore the value of including CPs in regulatory frameworks for ambient air pollutants in China and other rapidly industrializing regions.

## 4. Conclusions

These findings highlight the need for regional and international collaboration to address CP pollution. The transboundary nature of CP emissions demands stricter standards and sustainable industrial practices. Future research on gas-phase concentrations and indoor exposure can better assess their risks and guide effective mitigation strategies.

## Figures and Tables

**Table 1:** Concentrations of CPs in PM_1_ from the Pearl River Delta region, China.

	Median (ng/m^3^)	Geometric Mean (ng/m^3^)	Range	Cl (%)
Total				
ΣCPs	34	35.2	14.1–181.9	-
ΣSCCPs	17.3	18.5	8.7–89	60.8
ΣMCCPs	15	14.9	5.1–78.5	58.1
ΣLCCPs	0.7	0.6	0.02–16.4	56.7
Guangzhou				
ΣCPs	35	35.6	16.4–85.9	-
ΣSCCPs	17.3	19.3	8.8–62.1	60.8
ΣMCCPs	13.7	13.2	6.6–31.3	58.1
ΣLCCPs	0.9	0.7	0.02–4.9	51
Foshan				
ΣCPs	26.4	28.9	18–74	-
ΣSCCPs	14.9	16.5	10–36.5	60.8
ΣMCCPs	10.1	11.8	6.7–34.2	58.1
ΣLCCPs	0.3	0.3	0.03–3.3	56.9
Zhongshan				
ΣCPs	33.8	34.5	24.1–60.7	-
ΣSCCPs	16.2	16.6	10.9–27.8	60.8
ΣMCCPs	16.4	16.7	11.7–38	58.2
ΣLCCPs	0.9	0.9	0.3–2.5	56.6
Zhuhai				
ΣCPs	45.2	40.1	18.5–59.7	-
ΣSCCPs	20.7	20.9	9.8–31	60.5
ΣMCCPs	17.2	17.5	8.4–28.1	57.8
ΣLCCPs	0.8	0.5	0.05–2.3	48.6
Shenzhen				
ΣCPs	34.2	40	17.1–91.6	-
ΣSCCPs	18.6	18	9.88–39.3	60.9
ΣMCCPs	14.8	15.5	7.1–35.9	58.1
ΣLCCPs	0.8	0.9	0.1–16.4	56.8
Maoming				
ΣCPs	40.3	40.5	14.1–181.9	-
ΣSCCPs	18.3	20.8	8.7–89	60.5
ΣMCCPs	20.4	18.4	5.1–78.5	58.1
ΣLCCPs	0.6	0.6	0.1–14.4	56.8

## Data Availability

Data requests can be made to the first author.

## Supplementary Materials

The following supporting information can be downloaded at: https://www.mdpi.com/article/10.3390/toxics13060467/s1, Table S1. List of the chemicals and reagents for CPs analysis. Table S2. Quantification and qualification ions of SCCPs and MCCPs. Table S3. Quantification and qualification ions of LCCPs. Table S4. Site coordinates, air pressure and temperature of the sampling site in the primary and middle school from the PRD region, China. Table S5. Parameters for EDI calculations. Table S6. Overview of SCCP and MCCP concentrations (ng/m^3^) in ambient particles. Figure S1. Observed concentration and predicted concentration of SCCPs and LCCPs in all the PM1 samples. Figure S2. Concentrations of SCCP, MCCP, and LCCP in PM_1_ in the primary and middle schools of the six cities from the PRD region, China. Figure S3. The concentration of SCCPs, MCCPs, and LCCPs in PM_1_ in the primary and middle schools of the six cities from the PRD region, China. Figure S4. Source profiles of the finally retained seven-factor CPs solution. Factor 1: organic chemical industries; Factor 2: fugitive dust; Factor 3: sea salts; Factor 4: crustal dust; Factor 5: traffic source; Factor 6: metal smelting; Factor 7: secondary formation and combustion. Figure S5. Backward trajectories of air masses in the primary and middle schools of the six cities from the PRD region, China. Figure S6. Correlation between SCCPs, MCCPs, and LCCPs in PM1 in the primary and middle schools of the six cities from the PRD region, China. Figure S7. Concentrations of metals, carbon, anion, and cation in PM_1_ in the primary and middle schools of the six cities from the PRD region, China. Figure S8. Correlation between CP, SCCP, MCCP, LCCP and PM_1_, as well as other components (metals, cations, anions, carbon) within PM1 in the primary and middle schools of the six cities from the PRD region, China. Figure S9. Age-specific estimated daily intakes (EDI) and hazard quotients (HQ) of ΣCPs through PM_1_ from the six cities inhalation. Solid lines indicated the median of each group. Figure S10. Age-specific MOE of SCCPs, MCCPs, and LCCPs through PM_1_ inhalation. Solid lines indicated the median of each group.

## Abbreviations

BW	body weight
CP	chlorinated paraffin
EDI	estimated daily intake
HQ	hazard quotient
IR	inhalation rate
LCCP	long-chain chlorinated paraffin
LOQ	Limit of quantification
MCCP	medium-chain chlorinated paraffin
MOE	margin of exposure
NOAEL	non-observed adverse-effect level
PM	particulate matter
PMF	positive matrix factorization
PRD	Pearl River Delta
SCCP	short-chain chlorinated paraffin
TDI	tolerable daily intake
