# Supplementary material for: Distribution, Sources, and Health Risk of Short-, Medium- and Long-Chain Chlorinated Paraffins in School-Area Ambient PM1: A Study from the Pearl River Delta, China
Source: Toxics. 2025 May 31;13(6):467. doi: 10.3390/toxics13060467 (PMC12197006; doi:10.3390/toxics13060467)
Supplement: Supplementary file 1 [file toxics-13-00467-s001.zip › toxics-3647358-supplementary.pdf]

Supplementary Materials

Distribution, source, and health risk of short-, medium- and long-chain chlorinated paraffins in school ambient PM_1: A Study from the Pearl River Delta, China

Mo Yang, Xin-Feng Wang, Jing-Wen Huang, Nan-Xiang Jin, Chu Chu, Guo-Feng Huang, Duo-Hong Chen, Min Xie, Yu-Hong Zhai, Yu-Jun Lin, Jun Liu, Li-Zi Lin, Wen-Wen Bao, Zhao-Huan Gui, Pasi I. Jalava, Guang-Hui Dong, Marjut Roponen

Table of Contents

Table S1. List of the chemicals and reagents for CPs analysis

Table S2. Quantification and qualification ions of SCCPs and MCCPs.

Table S3. Quantification and qualification ions of LCCPs.

Table S4. Site coordinates, air pressure and temperature of the sampling site in the primary and middle school from the PRD region, China.

Table S5. Parameters for EDI calculations.

Table S6. Overview of SCCP and MCCP concentrations (ng/m³) in ambient particles.

Figure S1. Observed concentration and predicted concentration of SCCPs and LCCPs in all the PM_1 samples.

Figure S2. Concentrations of SCCP, MCCP, and LCCP in PM_1 in the primary and middle schools of the six cities from the PRD region, China.

Figure S3. The concentration of SCCPs, MCCPs, and LCCPs in PM_1 in the primary and middle schools of the six cities from the PRD region, China.

Figure S4. Source profiles of the finally retained seven-factor CPs solution. Factor 1: organic chemical industries; Factor 2: fugitive dust; Factor 3: sea salts; Factor 4: crustal dust; Factor 5: traffic source; Factor 6: metal smelting; Factor 7: secondary formation and combustion.

Figure S5. Backward trajectories of air masses in the primary and middle schools of the six cities from the PRD region, China.

Figure S6. Correlation between SCCPs, MCCPs, and LCCPs in PM_1 in the primary and middle schools of the six cities from the PRD region, China.

Figure S7. Concentrations of metals, carbon, anion, and cation in PM_1 in the primary and

middle schools of the six cities from the PRD region, China.

Figure S8. Correlation between CP, SCCP, MCCP, LCCP and PM_1, as well as other components (metals, cations, anions, carbon) within PM_1 in the primary and middle schools of the six cities from the PRD region, China.

Figure S9. Age-specific estimated daily intakes (EDI) and hazard quotients (HQ) of ΣCPs through PM_1 from the six cities inhalation. Solid lines indicated the median of each group.

Figure S10. Age-specific MOE of SCCPs, MCCPs, and LCCPs through PM_1 inhalation. Solid lines indicated the median of each group.

Table S1. List of the chemicals and reagents for CPs analysis

Chemical and reagents	Source
Standards of three SCCPs mixtures (chlorine contents of 51.5%, 55.5% and 63%)	Ehrenstorfer GmbH (Augsburg, Germany)
Standards of three MCCPs mixtures (chlorine contents of 42%, 52% and 57%)	Ehrenstorfer GmbH (Augsburg, Germany)
Standards of two LCCPs mixtures (chlorine contents of 36% and 49%)	Ehrenstorfer GmbH (Augsburg, Germany)
Surrogate standard 13C10-trans-chlordane	Cambridge Isotope Laboratories (Andover, USA)
Internal standards ε-hexachlorocyclohexane	Ehrenstorfer GmbH (Augsburg, Germany)
Internal standards 13C6-triclocarban	Toronto Research Chemicals (Toronto, Canada)
Silica gel (63-100 μm)	Merck (Whitehouse Station, USA)
Florisil (60-100 mesh)	Merck (Whitehouse Station, USA)
Anhydrous sodium sulfate	Merck (Whitehouse Station, USA)
Dichloromethane (HPLC)	Oceanpak (Sweden),<br>Fisher Scientific (Hanover Park, USA)
n-hexane (HPLC)	Oceanpak (Sweden),<br>Fisher Scientific (Hanover Park, USA)
Methanol (HPLC)	Oceanpak (Sweden),<br>Fisher Scientific (Hanover Park, USA)
Ammonium acetate (HPLC)	Oceanpak (Sweden),<br>Fisher Scientific (Hanover Park, USA)

Table S2. Quantification and qualification ions of SCCPs and MCCPs.

**SCCPs**	Quantification ion	Qualification ion	**MCCPs**	Quantification ion	Qualification ion
C10H18Cl4	277.0084	279.0055	C14H26Cl4	333.0710	335.0681
C10H17Cl5	312.9665	314.9636	C14H25Cl5	369.0291	371.0271
C10H16Cl6	346.9281	344.9310	C14H24Cl6	402.9907	404.9879
C10H15Cl7	380.8891	382.8862	C14H23Cl7	436.9518	438.9489
C10H14Cl8	416.8472	414.8502	C14H22Cl8	472.9099	470.9128
C10H13Cl9	450.8083	448.8112	C14H21Cl9	506.8709	504.8738
C10H12Cl10	484.7693	486.7664	C14H20Cl10	540.8319	542.829
C10H11Cl11	518.7297	520.7268	C14H19Cl11	574.7923	576.7894
C10H10Cl12	552.6908	554.6878	C14H18Cl12	608.7534	610.7504
C11H20Cl4	291.0241	293.0211	C14H17Cl13	642.7144	644.7114
C11H19Cl5	326.9822	328.9792	C15H28Cl4	347.0867	349.0837
C11H18Cl6	360.9438	362.9409	C15H27Cl5	383.0447	385.0418
C11H17Cl7	394.9048	396.9019	C15H26Cl6	417.0064	419.0035
C11H16Cl8	430.8629	428.8658	C15H25Cl7	450.9674	452.9645
C11H15Cl9	464.8239	462.8268	C15H24Cl8	486.9256	484.9284
C11H14Cl10	498.7849	500.7820	C15H23Cl9	520.8866	518.8895
C11H13Cl11	532.7454	534.7424	C15H22Cl10	554.8476	556.8447
C11H12Cl12	566.7064	568.7035	C15H21Cl11	588.8080	590.8050
C12H22Cl4	305.0397	307.0368	C15H20Cl12	622.7690	624.7661
C12H21Cl5	340.9978	342.9949	C15H19Cl13	656.7300	658.7271
C12H20Cl6	374.9594	376.9565	C16H30Cl4	361.1023	363.0994
C12H19Cl7	408.9205	410.9175	C16H29Cl5	397.0604	399.0574
C12H18Cl8	444.8786	442.8815	C16H28Cl6	431.0221	433.0192
C12H17Cl9	478.8396	476.8425	C16H27Cl7	464.9831	466.9802
C12H16Cl10	512.8006	514.7977	C16H26Cl8	500.9412	498.9441
C12H15Cl11	546.7610	548.7581	C16H25Cl9	534.9022	532.9051
C12H14Cl12	580.7221	582.7191	C16H24Cl10	568.8632	570.8604
C13H24Cl4	319.0554	321.0524	C16H23Cl11	602.8236	604.8207
C13H23Cl5	355.0135	357.0105	C16H22Cl12	636.7847	638.7817
C13H22Cl6	388.9751	390.9722	C16H21Cl13	670.7457	672.7427
C13H21Cl7	422.9361	424.9332	C17H32Cl4	375.1180	377.1150
C13H20Cl8	458.8942	456.8971	C17H31Cl5	411.0761	413.0731
C13H19Cl9	492.8552	490.8582	C17H30Cl6	445.0377	447.0349
C13H18Cl10	526.8163	528.8134	C17H29Cl7	478.9987	480.9959
C13H17Cl11	560.7767	562.7737	C17H28Cl8	514.9569	512.9598
C13H16Cl12	594.7377	596.7348	C17H27Cl9	548.9179	546.9208
-	-	-	C17H26Cl10	582.8789	584.876
-	-	-	C17H25Cl11	616.8393	618.8363
-	-	-	C17H24Cl12	650.8003	652.7974
-	-	-	C17H23Cl13	684.7613	686.7584

Table S3. Quantification and qualification ions of LCCPs.

	Quantification ion	Qualification ion		Quantification ion	Qualification ion		Quantification ion	Qualification ion
C18H34Cl4	389.1336	391.1307	C21H34Cl10	638.9409	640.9379	C24H36Cl14	818.8290	820.8260
C18H33Cl5	425.0913	427.0885	C21H33Cl11	672.9019	674.8989	C24H35Cl15	852.7900	854.7871
C18H32Cl6	459.0523	461.0495	C21H32Cl12	706.8629	708.8600	C25H48Cl4	487.2432	489.2402
C18H31Cl7	493.0133	495.0105	C21H31Cl13	740.8239	742.8210	C25H47Cl5	523.2013	525.1983
C18H30Cl8	528.9715	526.9743	C21H30Cl14	776.7820	778.7791	C25H46Cl6	557.1623	559.1594
C18H29Cl9	562.9325	560.9354	C22H42Cl4	445.1962	447.1933	C25H45Cl7	591.1233	593.1204
C18H28Cl10	596.8935	598.8906	C22H41Cl5	481.1543	483.1514	C25H44Cl8	625.0844	627.0814
C18H27Cl11	630.8549	632.8520	C22H40Cl6	515.1154	517.1124	C25H43Cl9	659.0454	661.0424
C18H26Cl12	664.8160	666.8130	C22H39Cl7	549.0764	551.0734	C25H42Cl10	695.0035	697.0005
C18H25Cl13	698.7770	700.7740	C22H38Cl8	583.0374	585.0344	C25H41Cl11	728.9645	730.9615
C18H24Cl14	734.7351	736.7321	C22H37Cl9	616.9984	618.9955	C25H40Cl12	762.9255	764.9226
C19H36Cl4	403.1493	405.1463	C22H36Cl10	652.9565	654.9536	C25H39Cl13	796.8865	798.8836
C19H35Cl5	439.1069	441.1042	C22H35Cl11	686.9175	688.9146	C25H38Cl14	832.8446	834.8417
C19H34Cl6	473.0680	475.0651	C22H34Cl12	720.8786	722.8756	C25H37Cl15	866.8057	868.8027
C19H33Cl7	507.0290	509.0261	C22H33Cl13	754.8396	756.8366	C26H50Cl4	501.2588	503.2559
C19H32Cl8	542.9871	540.9900	C22H32Cl14	790.7977	792.7947	C26H49Cl5	537.2169	539.2140
C19H31Cl9	576.9481	574.9510	C22H31Cl15	824.7587	826.7558	C26H48Cl6	571.1780	573.1750
C19H30Cl10	610.9091	612.9063	C23H44Cl4	459.2119	461.2089	C26H47Cl7	605.1390	607.1360
C19H29Cl11	644.8706	646.8676	C23H43Cl5	495.1700	497.1670	C26H46Cl8	639.1000	641.0971
C19H28Cl12	678.8316	680.8287	C23H42Cl6	529.1310	531.1281	C26H45Cl9	673.0610	675.0581
C19H27Cl13	712.7926	714.7897	C23H41Cl7	563.0920	565.0891	C26H44Cl10	709.0191	711.0162
C19H26Cl14	748.7507	750.7478	C23H40Cl8	597.0531	599.0501	C26H43Cl11	742.9801	744.9772
C20H38Cl4	417.1649	419.1620	C23H39Cl9	631.0141	633.0111	C26H42Cl12	776.9412	778.9382
C20H37Cl5	453.1226	455.1199	C23H38Cl10	666.9722	668.9692	C26H41Cl13	810.9022	812.8992
C20H36Cl6	487.0836	489.0808	C23H37Cl11	700.9332	702.9302	C26H40Cl14	846.8603	848.8573
C20H35Cl7	521.0446	523.0418	C23H36Cl12	734.8942	736.8913	C27H52Cl4	515.2745	517.2715
C20H34Cl8	557.0028	555.0056	C23H35Cl13	768.8552	770.8523	C27H51Cl5	551.2326	553.2296
C20H33Cl9	590.9638	588.9667	C23H34Cl14	804.8133	806.8104	C27H50Cl6	585.1936	587.1907
C20H32Cl10	624.9248	626.922	C23H33Cl15	838.7744	840.7714	C27H49Cl7	619.1546	621.1517
C20H31Cl11	658.8862	660.8833	C24H46Cl4	473.2275	475.2246	C27H48Cl8	653.1157	655.1127
C20H30Cl12	692.8473	694.8443	C24H45Cl5	509.1856	511.1827	C27H47Cl9	687.0767	689.0737
C20H29Cl13	726.8083	728.8053	C24H44Cl6	543.1467	545.1437	C27H46Cl10	723.0348	725.0318
C20H28Cl14	762.7664	764.7634	C24H43Cl7	577.1077	579.1047	C27H45Cl11	756.9958	758.9928
C21H40Cl4	431.1806	433.1776	C24H42Cl8	611.0687	613.0658	C27H44Cl12	790.9568	792.9539
C21H39Cl5	467.1387	469.1357	C24H41Cl9	645.0297	647.0268	C27H43Cl13	824.9178	826.9149
C21H38Cl6	501.0997	503.0967	C24H40Cl10	680.9878	682.9847	C27H42Cl14	860.8759	862.8730
C21H37Cl7	535.0607	537.0578	C24H39Cl11	714.9488	716.9459	C27H41Cl15	894.8370	896.8340
C21H36Cl8	569.0217	571.0188	C24H38Cl12	748.9099	750.9069			
C21H35Cl9	602.9828	604.9798	C24H37Cl13	782.8709	784.8679			

Table S4. Site coordinates, air pressure and temperature of the sampling site in the primary and middle school from the PRD region, China.

ID	City	Site coordinates	Air pres. (kPa)	Temp. (C^{o})
1	Guangzhou	(113.5203, 23.2598)	101.5	26.4
2	Guangzhou	(113.2546, 23.1374)	101.9	29.3
3	Guangzhou	(113.2546, 23.1374)	101.9	29.3
4	Guangzhou	(113.5782, 23.5505)	100.8	34.8
5	Guangzhou	(113.3579, 22.9259)	101.99	22.1
6	Guangzhou	(113.2418, 23.1458)	101.7	27.9
7	Guangzhou	(113.6520, 23.6482)	101.9	29.9
8	Guangzhou	(113.3547, 23.0842)	101.8	27.6
9	Guangzhou	(113.3298, 23.1315)	101.9	31
10	Guangzhou	(113.1988, 23.4028)	102	29.2
11	Guangzhou	(113.2677, 23.0951)	101.6	25.9
12	Guangzhou	(113.5705, 23.2808)	102	27.5
13	Guangzhou	(113.3400, 22.9163)	101.8	24.9
14	Guangzhou	(113.2794, 23.1420)	101.5	24.9
15	Guangzhou	(113.3279, 23.1022)	101.4	26.3
16	Guangzhou	(113.5485, 22.7105)	102	27.3
17	Guangzhou	(113.0724, 23.3924)	101.9	28.7
18	Guangzhou	(113.4409, 23.0991)	101.2	29.5
19	Guangzhou	(113.3537, 22.9094)	101.8	24.9
20	Guangzhou	(113.5441, 22.7058)	102.1	26.3
21	Guangzhou	(113.0155, 23.4399)	101.9	31.6
22	Guangzhou	(113.2557, 23.0925)	102	34.3
23	Guangzhou	(113.3137, 23.1409)	101.8	27.6
24	Guangzhou	(113.0616, 23.4409)	98.5	32
25	Guangzhou	(113.3230, 23.0999)	101.9	32.7
26	Guangzhou	(113.3509, 23.0707)	100.8	22
27	Guangzhou	(113.5827, 23.5569)	101.9	29.9
28	Guangzhou	(113.2550, 23.1272)	101.3	26.9
29	Guangzhou	(113.2526, 23.1071)	100.8	23
30	Maoming	(110.8996, 21.6650)	101.5	29.8
31	Maoming	(110.9379, 21.6522)	101.2	31.5
32	Maoming	(111.0251, 21.4739)	101	29.1
33	Maoming	(110.9361, 21.6527)	101.4	29
34	Maoming	(110.8914, 21.6937)	101.4	32.6
35	Maoming	(111.0270, 21.4725)	101.3	28.3
36	Maoming	(110.8439, 21.9050)	101.3	27.3
37	Maoming	(110.9092, 21.6628)	101.5	29.6
38	Maoming	(110.8932, 21.6633)	101.6	33.6
39	Maoming	(110.9021, 21.6571)	101.4	28.7
40	Maoming	(110.9099, 21.6552)	101.3	31.2
41	Maoming	(110.9231, 21.6738)	101.2	32.6

42	Maoming	(110.9226, 21.6637)	101.3	30.2
43	Maoming	(110.9215, 21.6693)	101.5	30.4
44	Foshan	(112.7864, 22.8746)	102.6	23.2
45	Foshan	(112.7698, 22.8633)	102.3	27.7
46	Foshan	(113.1120, 22.7259)	99.4	26.6
47	Foshan	(112.9003, 23.1516)	97.1	22.4
48	Foshan	(113.1378, 23.0013)	102	26.5
49	Foshan	(113.2688, 22.7735)	102	27.1
50	Foshan	(113.1300, 23.0560)	101.5	25.1
51	Foshan	(113.1113, 23.0273)	101.5	27.7
52	Foshan	(113.1206, 23.0363)	101.8	27.3
53	Foshan	(113.1214, 23.0298)	101.4	26.4
54	Foshan	(112.9004, 23.1641)	101.8	22.5
55	Foshan	(113.2407, 22.7574)	102.4	26.1
56	Foshan	(113.0918, 23.0398)	101.4	25.6
57	Foshan	(112.9213, 23.2005)	101.7	22.2
58	Zhuhai	(113.5487, 22.2320)	100.3	29.6
59	Zhuhai	(113.5267, 22.2726)	101.3	31.1
60	Zhuhai	(113.5245, 22.3738)	102	22
61	Zhuhai	(113.2919, 22.2039)	101.9	21.6
62	Zhuhai	(113.5436, 22.3783)	101.8	24.7
63	Zhuhai	(113.5556, 22.2670)	101.6	28.7
64	Zhuhai	(113.3564, 22.1738)	101.3	27.7
65	Zhongshan	(113.3978, 22.5260)	101.7	24.5
66	Zhongshan	(113.3755, 22.5075)	102.4	25.2
67	Zhongshan	(113.3725, 22.5399)	100.8	37.5
68	Zhongshan	(113.3891, 22.5177)	100.8	21.9
69	Zhongshan	(113.4443, 22.5021)	101.9	23.1
70	Zhongshan	(113.3737, 22.5135)	102	26.6
71	Zhongshan	(113.4167, 22.3360)	102	25.2
72	Zhongshan	(113.3709, 22.5368)	102	26.8
73	Zhongshan	(113.3925, 22.5400)	102.1	27.6
74	Zhongshan	(113.3691, 22.5249)	100.8	36.6
75	Zhongshan	(113.4821, 22.6177)	102.3	28.9
76	Zhongshan	(113.4926, 22.6280)	102.5	26.7
77	Zhongshan	(113.2173, 22.6895)	102.4	29
78	Zhongshan	(113.5177, 22.4948)	101.8	25
79	Zhongshan	(113.4241, 22.3531)	101.9	27.3
80	Zhongshan	(113.4318, 22.3454)	102.5	23.5
81	Zhongshan	(113.2224, 22.6692)	102.4	28.6
82	Zhongshan	(113.5241, 22.5050)	102.5	23.6
83	Shenzhen	(113.8906, 22.6741)	101.6	29.1
84	Shenzhen	(113.9363, 22.6872)	100.9	30.5
85	Shenzhen	(114.3110, 22.7756)	100.8	28.3

86	Shenzhen	(114.1153, 22.5627)	101.1	28.2
87	Shenzhen	(114.1213, 22.5632)	102.3	25.1
88	Shenzhen	(114.1388, 22.5598)	101.8	27.4
89	Shenzhen	(114.1288, 22.5870)	101.2	26.2
90	Shenzhen	(114.2979, 22.7750)	101.4	31
91	Shenzhen	(113.9089, 22.6608)	101.3	29.7
92	Shenzhen	(114.2970, 22.7695)	97.9	24.5
93	Shenzhen	(113.9217, 22.6507)	100.8	26.9

Table S5. Parameters for EDI calculations.

Age (y)	*IR* (m^3/d)	*BW* (kg)
<1[a]	4.9	8.3
1-2	5.7	11.2
2-3	6.3	13.5
3-4	8.0	15.6
4-5	8.4	17.7
5-6	8.8	19.6
6-9	10.1	26.5
9-12	13.2	36.8
12-15	13.5	47.3
15-18	14.0	54.8
18-44	16.7	61.9
45-59	16.7	63.5
60-79	13.8	60.3
80+	12.0	55.5

[a]: *IR* and *BW* of age < 1 y were the average of *IR* and *BW* provided of four age group (0-3 m, 3-6 m, 6-9 m and 9 m-1 y).

Table S6. Overview of SCCP and MCCP concentrations (ng/m^3) in ambient particles.

Sampling site	Sampling time	Sample type	Sampler	Analytical Instrument	SCCP			MCCP			Ref.
					**Median**	**Mean**	**Range**	**Median**	**Mean**	**Range**	
China (Pearl River Delta)	2018.10–2018.12 (winter)	PM$_1$	Med-vol (QFF)	LC-ESI-HRMS	17.3	18.5	8.7-89	15	14.9	5.1-78.5	This study
China (Pearl River Delta)	2018.5–2018.7 (summer)	PM$_{2.5}$	Med-vol (QFF)	LC-ESI-HRMS	17.62	23.0	5.36–109	20.8	26.0	4.4-109.6	(Huang et al. 2023)[1]
	2018.10–2018.12 (winter)		Hi-vol (QFF)		6.53	7.68	0.83–32.1	6.3	7.1	1.0-24.1	
China (Beijing)	2016.2–2016.5	PM$_{10}$	Low-vol (QFF)	GC-ECNI-qTOF		23.9	16.9–28.8		3.6	2.2-5.0	(Huang et al. 2017)[2]
		PM$_{2.5}$				14.9	9.2–19.6		1.7	0.6-2.7	
		PM$_{1.0}$				10.4	4.1–15.4		1	0.3-1.3	
China (Pearl River Delta)	2017.6–2017.8	PM$_{2.5}$	Hi-Vol (QFF)	GC-ECNI-LRMS			1.6–32.5			4.5-180	(Zhuo et al. 2019)[3]
China (Shandong)	2016.1–2016.11 (total)	PM$_{2.5}$	Med-vol (QFF)	GC-ECNI-LRMS		38.7	9.80–105				(Li et al. 2019)[4]
	2016.3–2016.5 (spring)					37.7	21.1–69.9				
	2016.6–2016.8 (summer)					29.7	9.80–45.3				
	2016.9–2016.11 (autumn)					32.8	10.1–46.4				
	2016.1–2016.2 (winter)					54.8	27.0–105				
China (10 cities)	2013.10–2014.8	PM$_{2.5}$	Hi-vol (QFF)	GC-ECNI-LRMS		19.9	1.98–274		15.6	1.3-312	(Liu et al. 2020)[5]
China (Henan)	2017.7–2018.10	Coarse	Med-vol (QFF+GFF)	GC-ECNI-Q-Orbitrap		5.20	3.55–359		1.6		(Li et al. 2021)[6]
		Fine				19.9			5.3		
		Ultrafine				6.93			2.3		

Low-vol: Low-volume sampler. Med-vol: Medium-volume sampler. Hi-vol: High-volume sampler. GFF: Glass fiber filter. QFF: Quartz fiber filter. PUF: Polyurethane foam.

References

1. Huang, J.; Zhao, L.; Shi, Y.; Zeng, X.; Sun, W.; Zhao, X.; Liu, R.; Wu, Q.; Dong, G.; Chen, D., Characterization of short-, medium-and long-chain chlorinated paraffins in ambient PM2. 5 from the Pearl River Delta, China. *Environment International* **2023,** *175*, 107932.

2. Huang, H.; Gao, L.; Xia, D.; Qiao, L.; Wang, R.; Su, G.; Liu, W.; Liu, G.; Zheng, M., Characterization of short-and medium-chain chlorinated paraffins in outdoor/indoor PM10/PM2. 5/PM1. 0 in Beijing, China. *Environmental Pollution* **2017,** *225*, 674-680.

3. Zhuo, M.; Ma, S.; Li, G.; Yu, Y.; An, T., Chlorinated paraffins in the indoor and outdoor atmospheric particles from the Pearl River Delta: Characteristics, sources, and human exposure risks. *Science of the Total Environment* **2019,** *650*, 1041-1049.
4. Li, H.; Li, J.; Li, H.; Yu, H.; Yang, L.; Chen, X.; Cai, Z., Seasonal variations and inhalation risk assessment of short-chain chlorinated paraffins in PM2. 5 of Jinan, China. *Environmental Pollution* **2019,** *245*, 325-330.
5. Liu, D.; Li, Q.; Cheng, Z.; Li, K.; Li, J.; Zhang, G., Spatiotemporal variations of chlorinated paraffins in PM2. 5 from Chinese cities: Implication of the shifting and upgrading of its industries. *Environmental Pollution* **2020,** *259*, 113853.
6. Li, Q.; Guo, M.; Song, H.; Cui, J.; Zhan, M.; Zou, Y.; Li, J.; Zhang, G., Size distribution and inhalation exposure of airborne particle-bound polybrominated diphenyl ethers, new brominated flame retardants, organophosphate esters, and chlorinated paraffins at urban open consumption place. *Science of the Total Environment* **2021,** *794*, 148695.

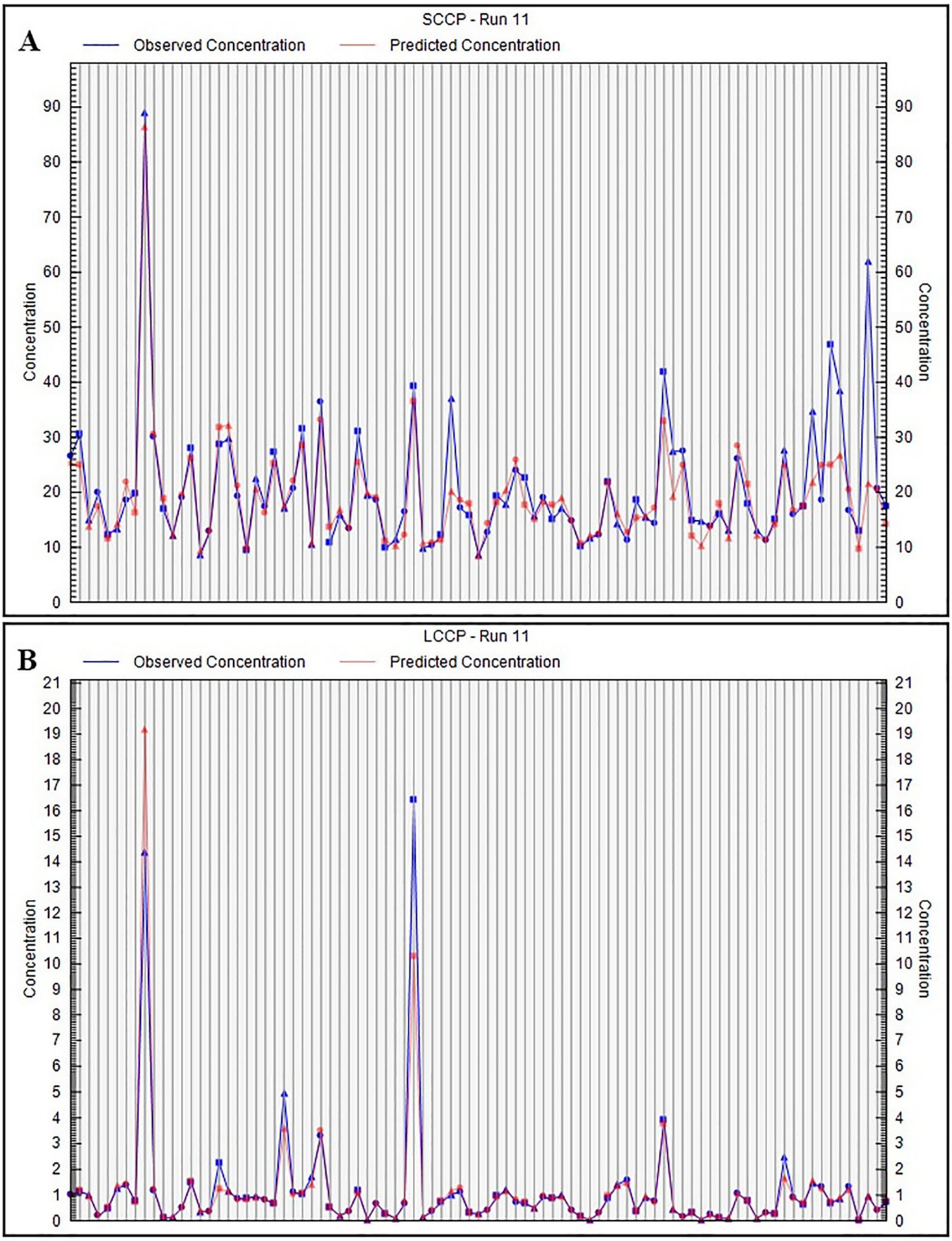

Figure S1. Observed concentration and predicted concentration of SCCPs and LCCPs in all the PM_1 samples.

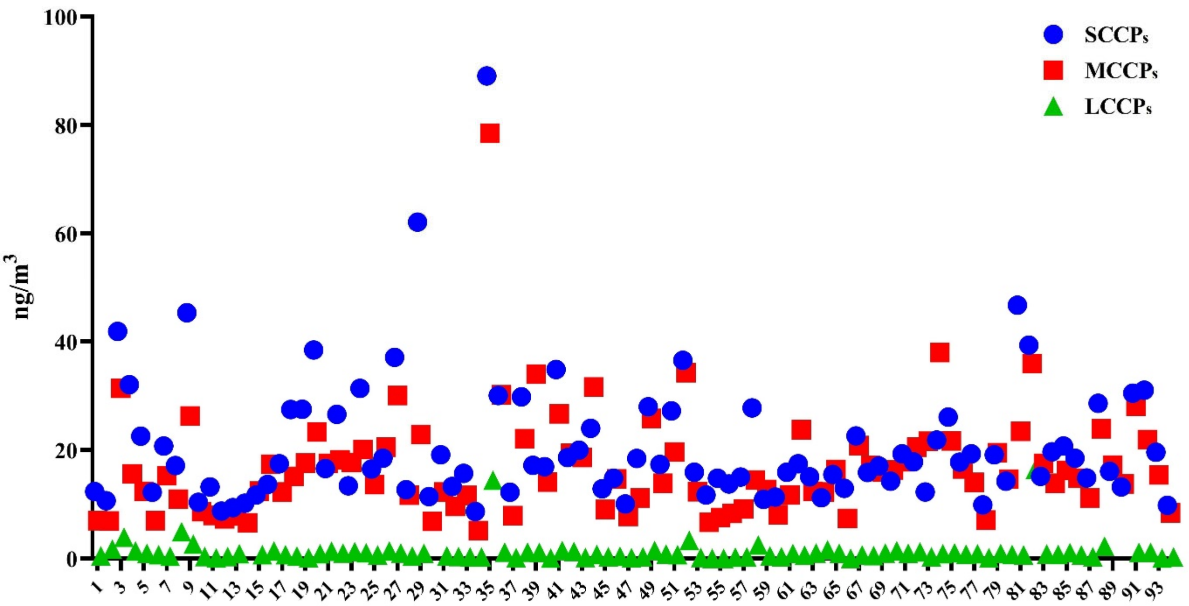

Figure S2. Concentrations of SCCP, MCCP, and LCCP in PM_1 in the primary and middle schools of the six cities from the PRD region, China.

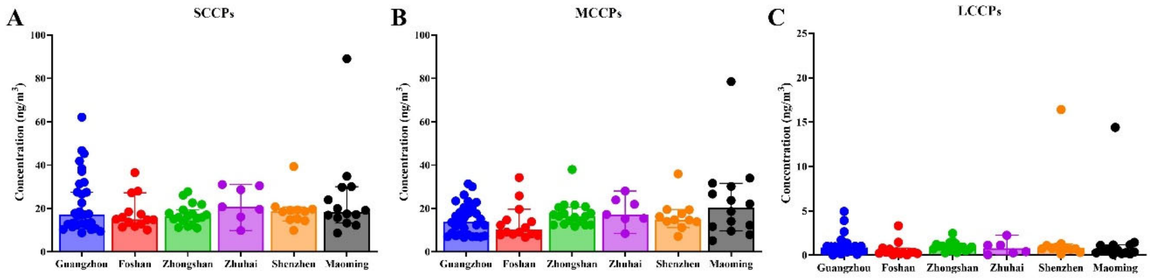

Figure S3. The concentration of SCCPs, MCCPs, and LCCPs in PM_1 in the primary and middle schools of the six cities from the PRD region, China.

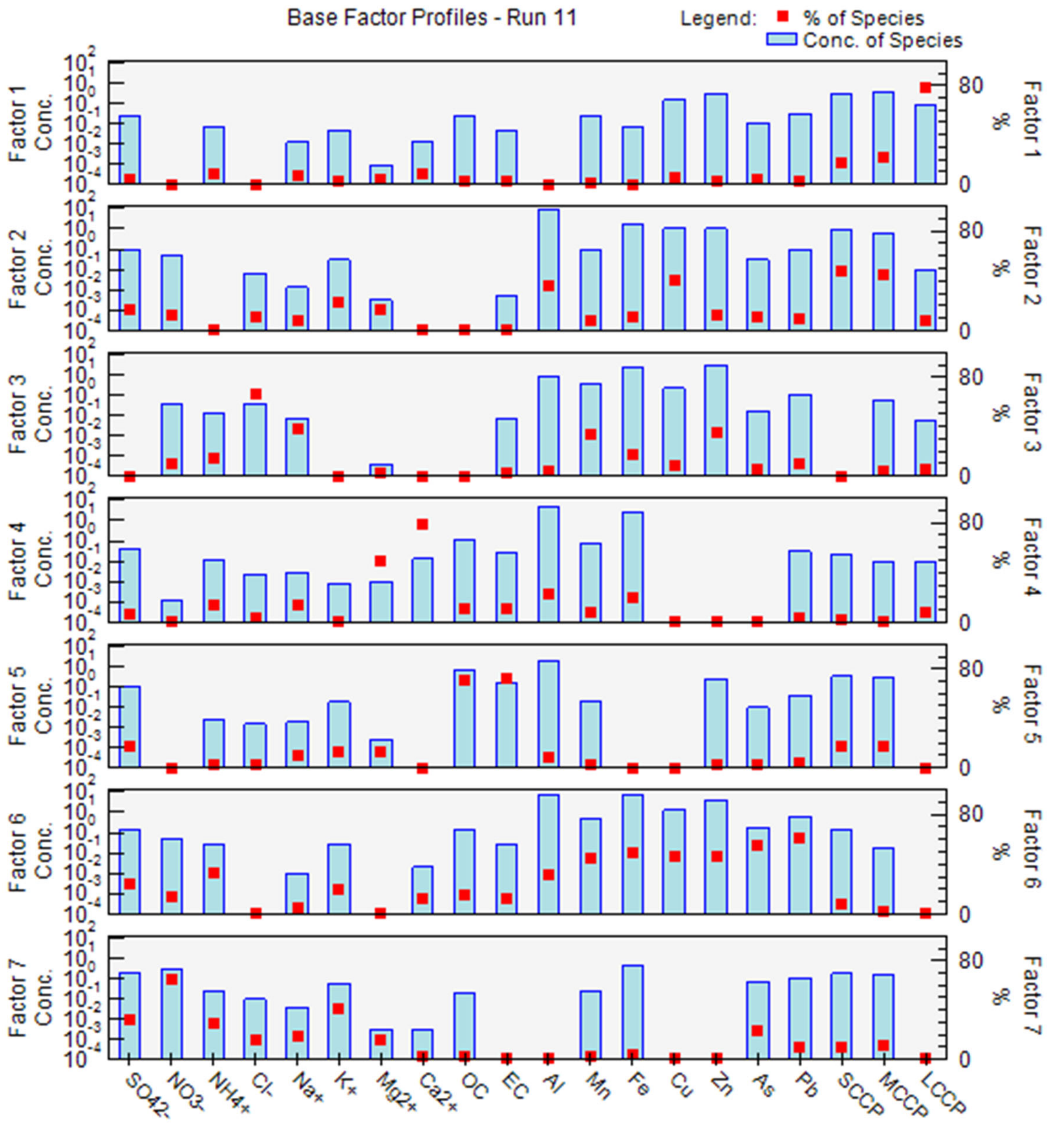

Figure S4. Source profiles of the finally retained seven-factor CPs solution. Factor 1: organic chemical industries; Factor 2: fugitive dust; Factor 3: sea salts; Factor 4: crustal dust; Factor 5: traffic source; Factor 6: metal smelting; Factor 7: secondary formation and combustion.

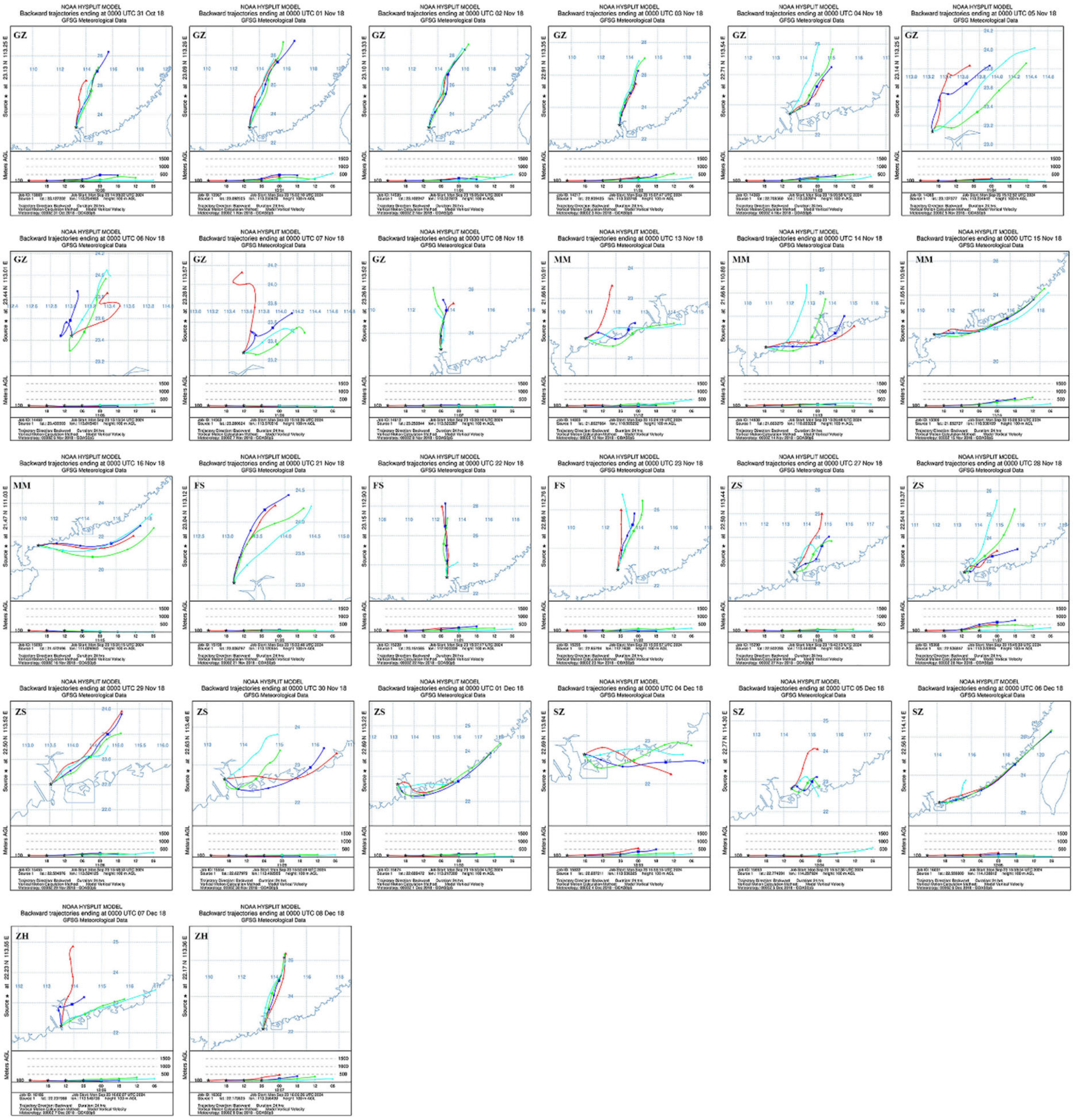

Figure S5. Backward trajectories of air masses in the primary and middle school of the six cities from the PRD region, China.

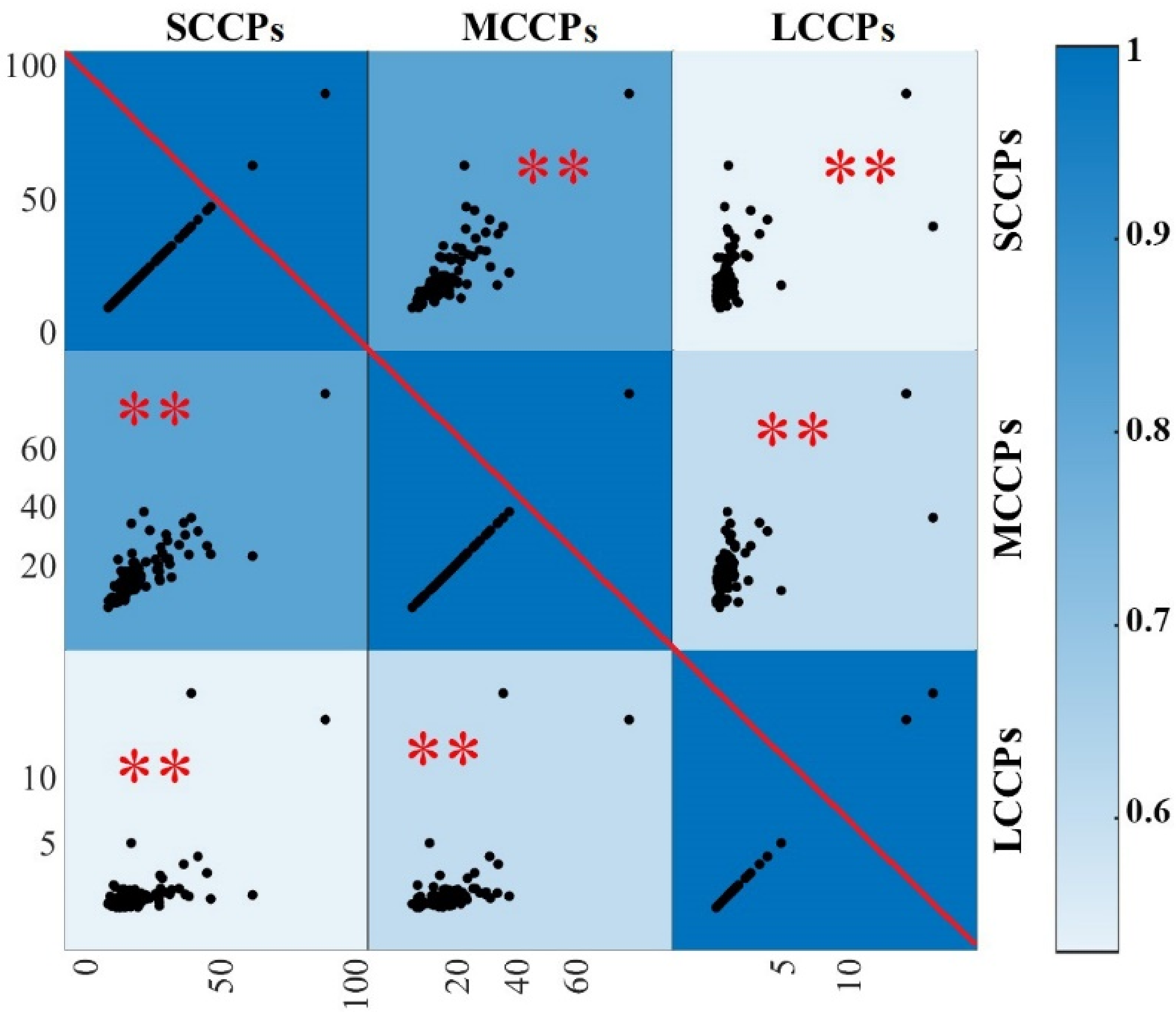

Figure S6. Correlation between SCCPs, MCCPs, and LCCPs in PM_1 in the primary and middle schools of the six cities from the PRD region, China. **: $P \leq 0.001$.

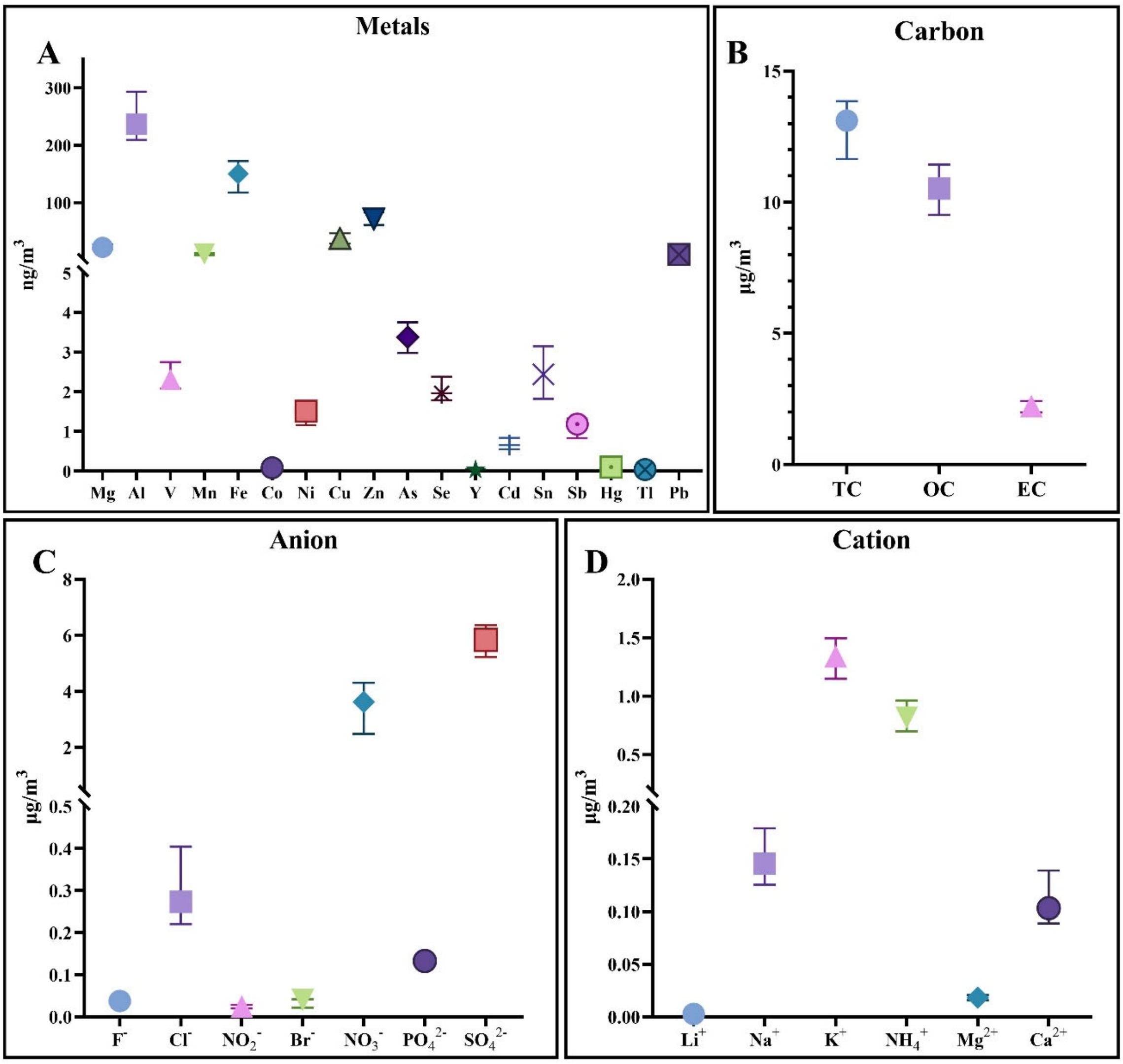

Figure S7. Concentrations of metals, carbon, anion, and cation in PM_1 in the primary and middle schools of the six cities from the PRD region, China.

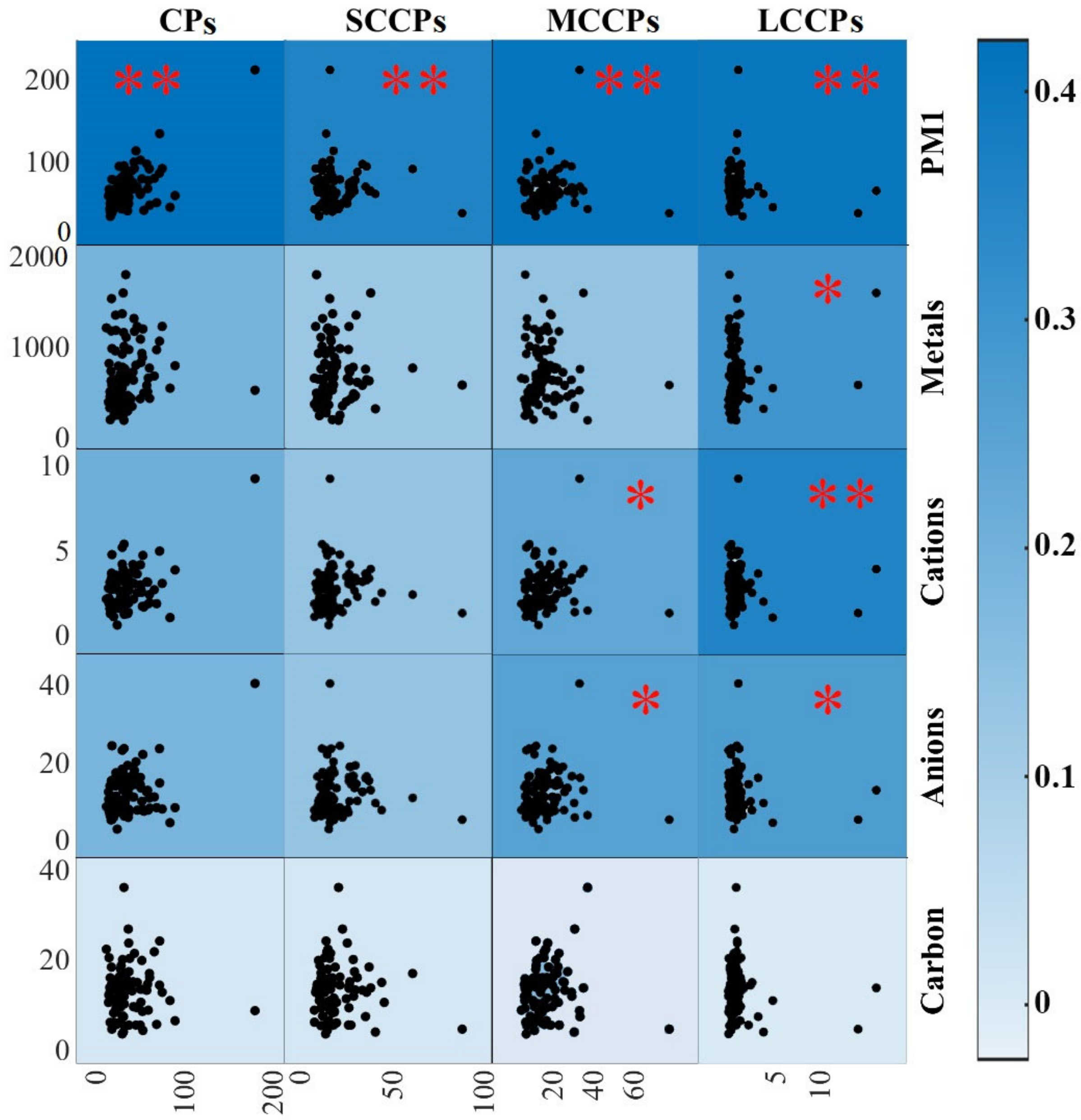

Figure S8. Correlation between CP, SCCP, MCCP, LCCP and PM_1, as well as other components (metals, cations, anions, carbon) within PM_1 in the primary and middle schools of the six cities from the PRD region, China. *: $P < 0.05$; **: $P \leq 0.001$.

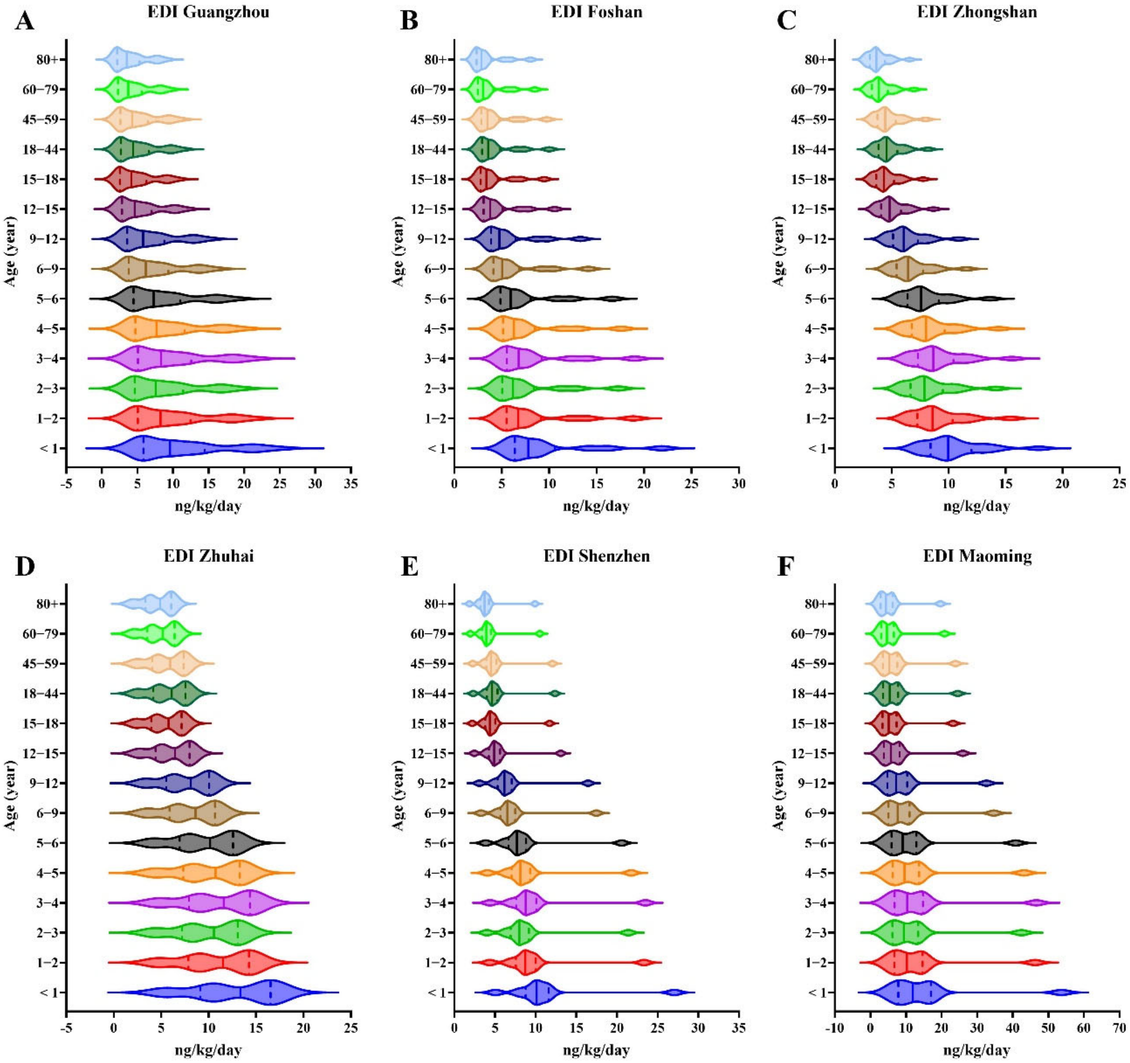

Figure S9. Age-specific estimated daily intakes (EDI) and hazard quotients (HQ) of ΣCPs through PM_1 from the six cities inhalation. Solid lines indicated the median of each group.

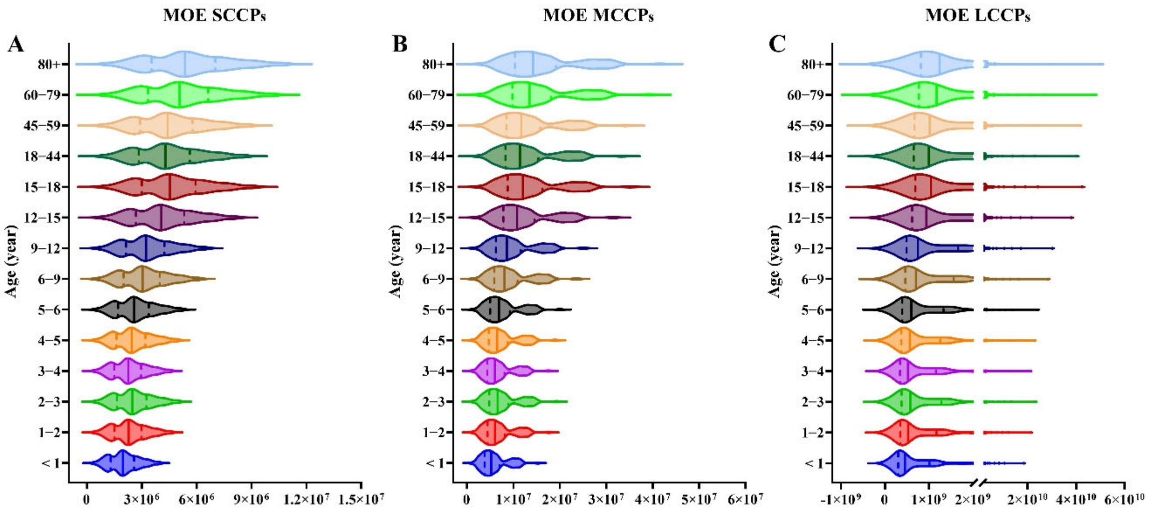

Figure S10. Age-specific MOE of SCCPs, MCCPs, and LCCPs through PM_1 inhalation. Solid lines indicated the median of each group.
